# A Compact Multi-Mode Reconfigurable Filter with Coarse- and Fine-Tunable Pole–Zero Characteristics for Ku/Ka Multi-Band Applications

**DOI:** 10.3390/mi17101150

**Published:** 2026-09-30

**Authors:** Jihan Fu, Xu Wang, Jincai Wen

**Affiliations:** Key Laboratory of Radio Frequency Circuits and Systems, Ministry of Education, Hangzhou Dianzi University, Hangzhou 310018, China

**Keywords:** multi-mode filter, Ku/Ka-band, pole–zero tracking, continuously tunable

## Abstract

This work presents a compact, multi-mode reconfigurable filter fabricated in a 65-nm CMOS process for Ku-/Ka multiband applications. The filter employs lumped-element LC series and parallel resonant networks to realize two transmission zeros (TZs) and two poles, with the TZs intrinsically clamped between the poles. Coarse-tuning is achieved by controlling voltage-controlled switches, enabling wide-range relocation of the pole–zero frequencies, while voltage-controlled varactors provide continuous fine-tuning. This pole–zero tracking mechanism allows the filter to suppress high-frequency image signals in Ku-band satellite communication terminals and low-frequency image signals in Ka-band applications. Measurements demonstrate 15-dB stopband fractional bandwidths of 58% and 58.3% in the coarse-low and coarse-high modes, respectively, with maximum attenuations exceeding 33 and 40 dB. Fine-tuning shifts the stopband center frequencies continuously from 14.5–19.7 GHz and 24.1–26.9 GHz, respectively, in close agreement with electromagnetic simulations. The measurement results confirm its suitability for multi-band and tunable image-rejection applications.

## 1. Induction

Recent advancements in 5G millimeter-wave (mmW) Frequency Range 2 (FR2) communications (24.25–52.6 GHz) require RF front ends capable of supporting high-bandwidth communications, while Ku-band satellite terminals (10.7–12.75 GHz for the downlink and 13.75–14.5 GHz for the uplink) remain indispensable in satellite communications. With the integration of these two communication systems, the radio frequency front end (RFFE) faces severe challenges from complex image frequency interference. Specifically, it must suppress both the high-frequency image signals generated in the Ku-band and the low-frequency image signals within the 5G Ka-band. Furthermore, tunable bandstop filters (BSFs), offering continuous frequency tunability, are needed for dynamic spectrum access and spectrum tuning issues. Consequently, investigating multi-mode reconfigurable filters that feature both dual-image suppression and continuous tunability has become a critical endeavor in RFFE design.

Over the past few decades, BSFs have been extensively investigated. In [1], a binary bandwidth-reconfigurable BSF incorporating both lumped- and distributed-element topologies was proposed, where 2^M^ discrete bandwidth states were achieved using M control voltages, and digital bandwidth reconfigurability was verified at 285 MHz and 2 GHz. In [2], a tunable BSF based on yttrium iron garnet (YIG)-micromachined technology was presented, maintaining a stopband rejection of over 30 dB across a 4–8 GHz tuning range, with a maximum attenuation of 55 dB. However, these BSFs employ off-chip technologies. Compared with off-chip filters, on-chip solutions are much more suitable for integration due to their miniaturized size, reduced fabrication cost, and enhanced frequency tuning precision. Based on standard silicon technologies, several innovative designs have been presented in the literature, which includes lowpass [3,4], highpass [5,6], bandpass [7,8,9,10,11,12,13,14], and bandstop filters (BSFs) [15,16,17,18,19]. Additional examples include a GaAs IPD bandstop filter [20], VO_2_-based reconfigurable millimeter-wave filters [21], a SiGe BiCMOS bandpass filter [22], and a MEMS-based tunable bandstop filter [23]. Collectively, these works provide context for evaluating the proposed CMOS implementation across different material platforms and fabrication technologies.

**Figure 1:**
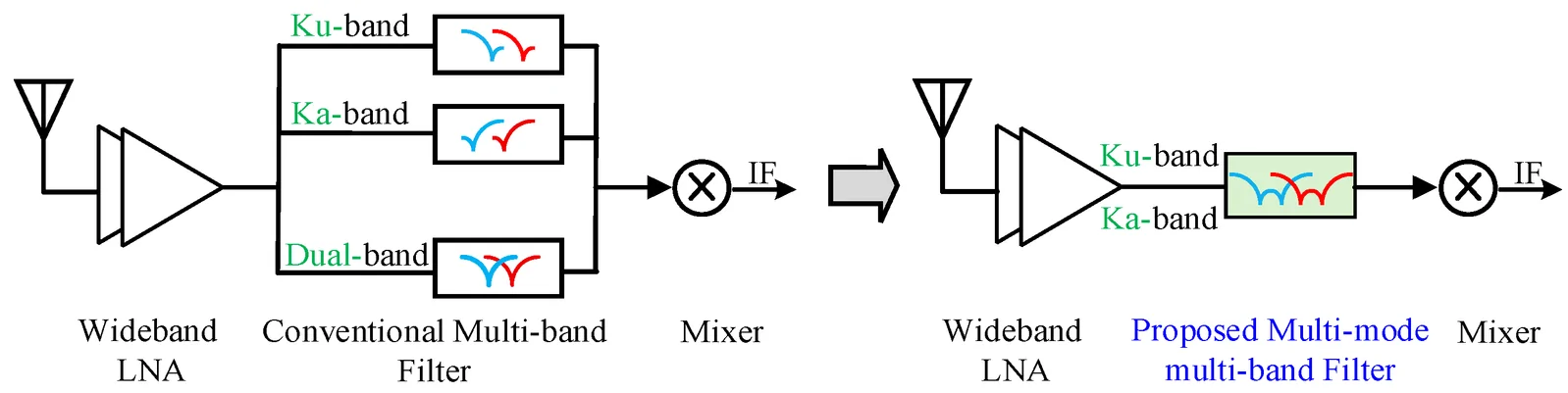
Block diagrams of the conventional and the proposed multi-band multi-mode filter receiver architectures.

Figure 1 illustrates the block diagrams of the conventional and the proposed multi-band multi-mode filter architectures. To effectively suppress the high-frequency image signals of the Ku-band and the low-frequency image signals of the Ka-band, conventional multi-mode reconfigurable filters typically require the integration of a low-frequency (LF) filter, a high-frequency (HF) filter, and a BSF between the low-noise amplifier (LNA) and the mixer. This approach not only increases the chip area but also leads to hardware resource redundancy. To address these issues, in this article, a compact multi-mode reconfigurable filter configuration is proposed for Ku/Ka multi-band applications using 65-nm CMOS technology. The proposed filter exhibits two transmission zeros (TZs) and two transmission poles (TPs). Additionally, it incorporates a pole–zero tracking mechanism, ensuring that the two TZs are inherently clamped between the TPs based on lumped-element LC series and parallel resonant networks. By controlling the on/off states of voltage-controlled switches, a wide-range coarse-tuning of the pole and zero frequencies is achieved, while the continuous adjustment of voltage-controlled varactors enables dynamic fine-tuning. Leveraging these two flexible TZs, the filter effectively suppresses image signals over a wide frequency range. The measured results are in good agreement with simulations, demonstrating that the proposed filter achieves a compact size, dual-image suppression, and frequency tunable features.

## 2. Design and Implementation of a Multi-Mode Reconfigurable Filter

**Figure 2:**
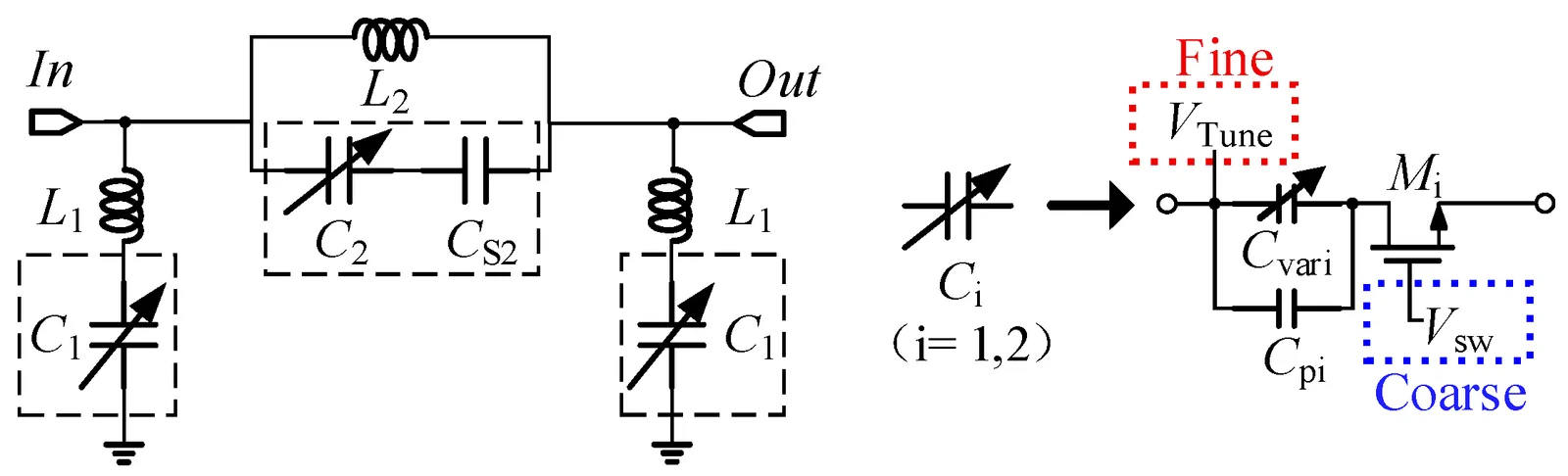
Schematic diagram of the proposed multi-mode reconfigurable filter.

Figure 2 presents the schematic diagram of the proposed compact multi-mode reconfigurable filter. To facilitate analysis and design, a symmetrical Π-type topology is adopted, which comprises two sets of LC series resonant networks and a central LC parallel resonant network. To achieve effective suppression of the image frequencies over a wide frequency band, the core mechanism of this circuit utilizes the resonant networks formed by inductors and tunable capacitors to introduce two TZs in the transfer function. Specifically, in this design, two tuning mechanisms, namely coarse-tuning and fine-tuning, are explored to control the pole and zero frequencies. Wherein, the coarse-tuning is realized by adjusting the control voltage *V*_sw_ to switch the state of the switch between on and off, allowing for a wide-range adjustment. Furthermore, the fine-tuning, together with the dynamic tracking capability, is achieved by varying the tuning voltage *V*_tune_ to dynamically adjust the capacitance of the varactor (*C*_var_).

### 2.1. Pole–Zero Generation and Distribution

To reveal the operating mechanism of the multi-mode reconfigurable filter. Based on the schematic equivalent model shown in Figure 2, this article employs Z-parameter analysis to evaluate the system’s pole–zero distribution.

First, To further elucidate the pole–zero distribution of the proposed filter topology, the transfer impedance *Z*_21_, neglecting the parasitic resistances, can be obtained as follows:

(1) $$Z_{21}=\frac{C_{2}{\left(\frac{1}{j\omega C_{1}}+j\omega L_{1}\right)}^{2}\left(\frac{1}{j\omega C_{2}}+j\omega L_{2}\right)}{2C_{2}\left(\frac{1}{j\omega C_{1}}+j\omega L_{1}\right)\left(\frac{1}{j\omega C_{2}}+j\omega L_{2}\right)+L_{2}}$$

By setting |Z_21_| = 0, the transmission zeros of the circuit can be derived as follows:

(2) $$\omega_{z1}=\frac{1}{\sqrt{L_{1}C_{1}}}$$

(3) $$\omega_{z2}=\frac{1}{\sqrt{L_{2}C_{2}}}$$

As indicated by (2) and (3), the zero frequencies of |Z_21_| are determined by the values of *C*_1_, *L*_1_, *C*_2_, and *L*_2_. Specifically, when *L*_1_*C*_1_ = *L*_2_*C*_2_, the topology exhibits only a single transmission zero. Conversely, when *L*_1_*C*_1_ ≠ *L*_2_*C*_2_, two transmission zeros are generated in the transmission response. According to (2) and (3), these two zeros are independent of *L*_2_,*C*_2_ and *L*_1_,*C*_1_, respectively.

Similarly, by setting |Z_21_| = ∞, the poles of |Z_21_| can be derived as follows:

(4) $$\omega_{p1}=\frac{1}{2}\sqrt{\frac{2L_{1}C_{1}+L_{2}C_{1}+2L_{2}C_{2}+\sqrt{-16L_{1}C_{1}L_{2}C_{2}+{\left(2L_{2}C_{2}+C_{1}\left(2L_{1}+L_{2}\right)\right)}^{2}}}{L_{1}C_{1}L_{2}C_{2}}}$$

(5) $$\omega_{p2}=\frac{1}{2}\sqrt{\frac{2L_{1}C_{1}+L_{2}C_{1}+2L_{2}C_{2}-\sqrt{-16L_{1}C_{1}L_{2}C_{2}+{\left(2L_{2}C_{2}+C_{1}\left(2L_{1}+L_{2}\right)\right)}^{2}}}{L_{1}C_{1}L_{2}C_{2}}}$$

**Figure 3:**
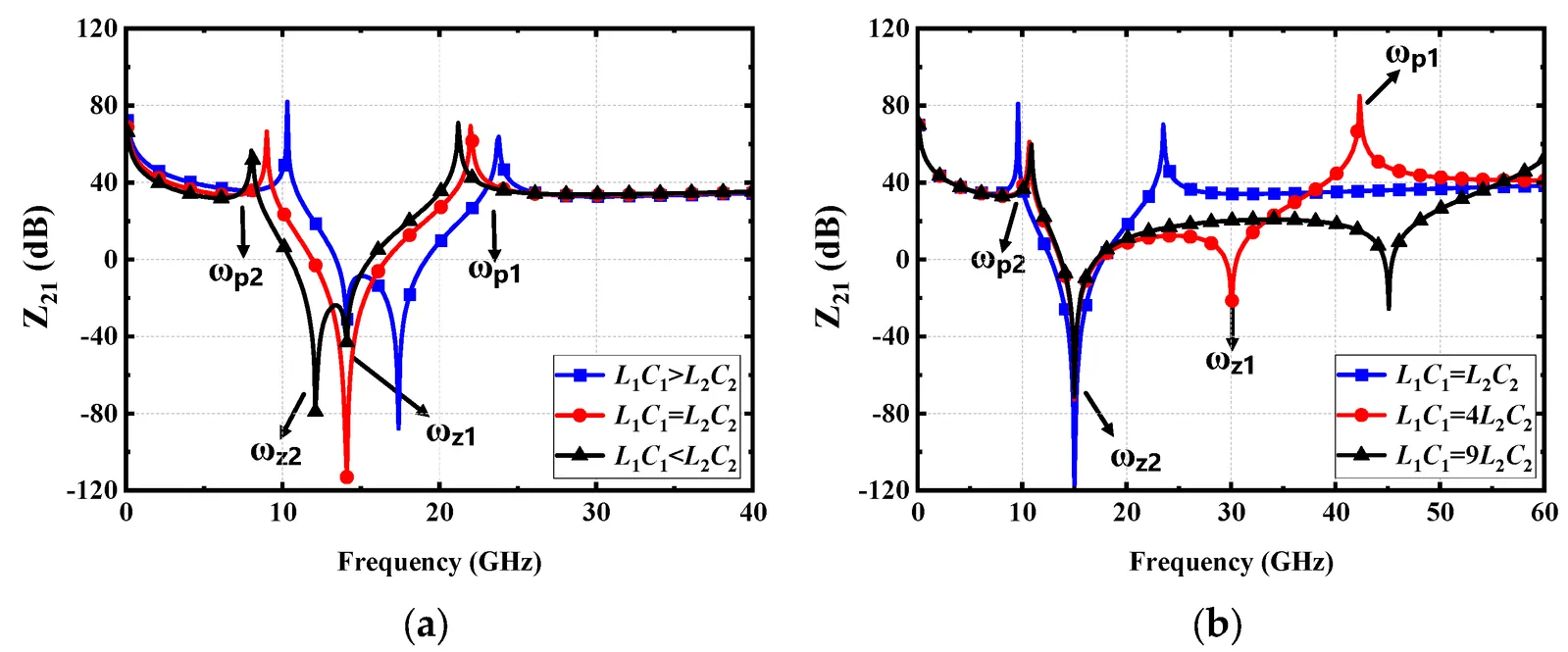
Simulated |Z_21_| curves of the proposed multi-mode reconfigurable filter for different (**a**) relationships between *L*_1_*C*_1_ and *L*_2_*C*_2_ and (**b**) ratios of *L*_1_*C*_1_ to *L*_2_*C*_2_.

Based on the aforementioned pole–zero analysis, the proposed structure is capable of generating two poles and two zeros in its transmission response. To validate this analysis, Figure 3 illustrates the simulated |Z_21_| curves of the proposed multi-mode reconfigurable filter under various conditions regarding the magnitude and ratio of *L*_1_*C*_1_ to *L*_2_*C*_2_. As shown in Figure 3a, when *L*_1_*C*_1_ = *L*_2_*C*_2_, the two zeros coincide, resulting in a single transmission zero. Conversely, when *L*_1_*C*_1_ ≠ *L*_2_*C*_2_, two distinct zeros emerge, which are consistently situated between the two poles. Furthermore, Figure 3b demonstrates that the frequency spacing between these two zeros is governed by the ratio of L_1_C_1_ to L_2_C_2_. Consequently, in practical design, this ratio can be flexibly adjusted to achieve the required bandwidth of the notched band.

### 2.2. Pole–Zero Tunable Characteristics

Building upon the detailed analysis of the pole–zero distribution in the preceding section, this section focuses on elucidating the relative positioning of the poles and zeros, as well as the continuous tunability of the filter.

Taking pole *ω*_p2_ and zero *ω*_Z1_ as examples, we examine the relative relationship between the pole and the zero within the structure. The expressions for *ω*_p2_ and *ω*_Z1_ are given in (5) and (2), respectively. This section aims to demonstrate that the ratio of *ω*_p2_ to *ω*_Z1_ is consistently less than unity. To this end, the variables are first normalized, and after performing the initial variable substitution, the expression for the ratio of *ω*_p2_ to *ω*_Z1_ is derived as (7):

(6) $$m=L_{1}C_{1},r=\frac{L_{2}}{L_{1}},k=\frac{C_{2}}{C_{1}}$$

(7) $$\frac{\omega_{P2}}{\omega_{z1}}=\frac{1}{2}\sqrt{\frac{2+r+2\mathrm{kr}-\sqrt{{\left(2+r+2\mathrm{kr}\right)}^{2}-16\mathrm{kr}}}{\mathrm{kr}}}$$

Subsequently, by performing a second variable substitution, the expression for the ratio of *ω*_p2_ to *ω*_Z1_ is derived as (9):

*p* = 2 + *r* + 2*kr* > 0,*q* = 4*kr* > 0 (8)

(9) $$\frac{\omega_{P2}}{\omega_{z1}}=\sqrt{\frac{p-\sqrt{p^{2}-4q}}{q}}$$

Analytical calculations confirm that Equation (10) holds universally for both p ≤ q and p > q.

(10) $$p-\sqrt{p^{2}-4q}<q;p,q>0$$

Based on the preceding analysis, the ratio *ω*_p2_/*ω*_Z1_ is consistently less than unity, indicating that *ω*_p2_ < *ω*_Z1_. By extension, it can be demonstrated that the low-frequency pole *ω*_p2_ is always lies below both zero frequencies *ω*_Z1_ and *ω*_Z2_, while the high-frequency pole *ω*_p1_ remains greater than both zeros, as summarized in Equations (11) and (12).

*ω_P_*_1_ > *ω_z_*_1_,*ω_P_*_1_ > *ω_z_*_2_;*C_i_*,*L_i_* > 0 (11)

*ω_P_*_1_ > *ω_z_*_1_,*ω_P_*_1_ > *ω_z_*_2_;*C_i_*,*L_i_* > 0 (12)

The robustness of the proposed mechanism originates from the pole–zero arrangement established by the symmetrical Π-type topology. As demonstrated by the preceding analysis and confirmed by (11) and (12), the two transmission zeros consistently remain between the two transmission poles for arbitrary inductance and capacitance values. Consequently, a wide stopband bounded by the lower and upper passbands is preserved. Although the exact pole and zero frequencies depend on the inductance and capacitance values, the fundamental bandstop mechanism remains unchanged as these parameters vary.

Having established the relative positioning of the zeros and poles in the transmission characteristics, we apply the substitution method used in Equation (6) to derive the ratios of the two pole frequencies of |Z21| to the two zero frequencies, as follows:

(13) $$\frac{\omega_{P1,P2}}{\omega_{z1}}=\frac{1}{2}\sqrt{\frac{2+r+2\mathrm{kr}\pm \sqrt{{\left(2+r+2\mathrm{kr}\right)}^{2}-16\mathrm{kr}}}{\mathrm{kr}}}$$

(14) $$\frac{\omega_{P1,P2}}{\omega_{z2}}=\frac{1}{2}\sqrt{2+r+2\mathrm{kr}\pm \sqrt{{\left(2+r+2\mathrm{kr}\right)}^{2}-16\mathrm{kr}}}$$

**Figure 4:**
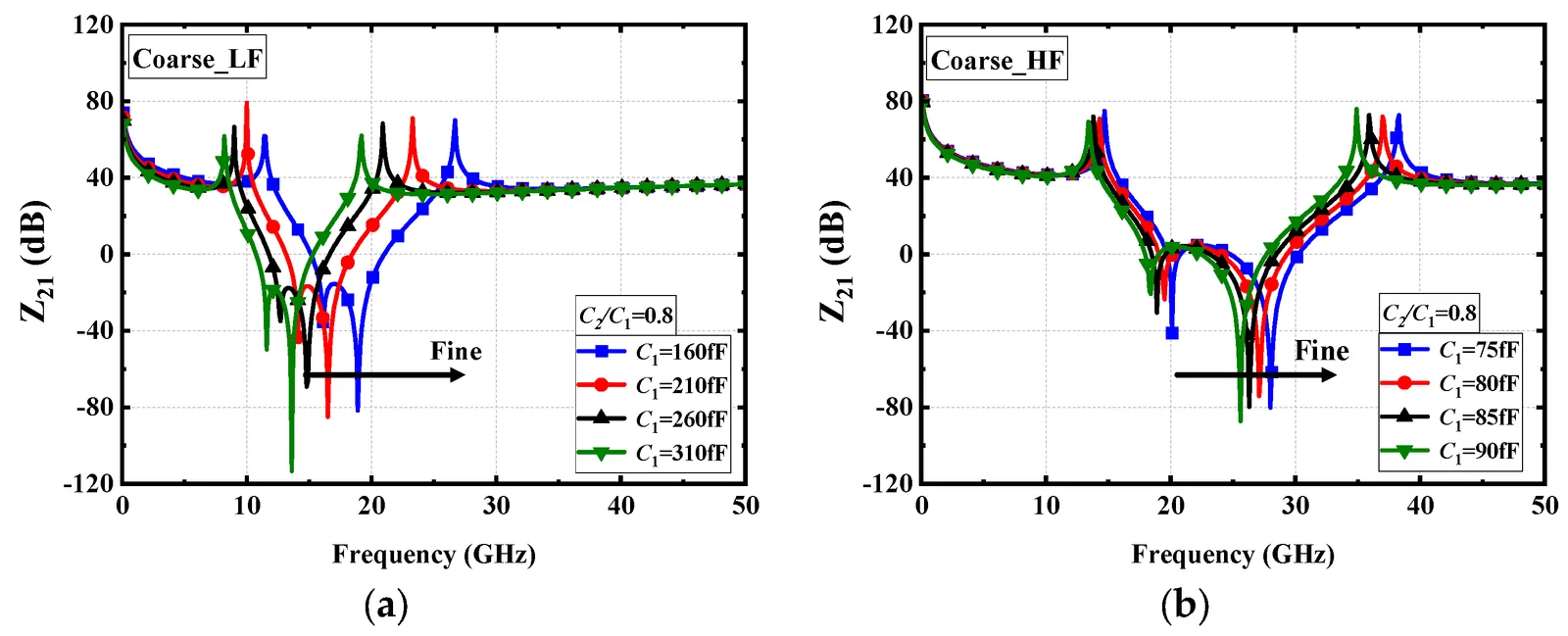
Simulated *Z*_21_ curves of the proposed multi-mode reconfigurable filter with k = 0.8 as C1 varies in (**a**) Coarse_LF mode and (**b**) Coarse_HF mode.

As indicated by Equations (13) and (14), the ratios of the pole and zero frequencies are primarily determined by the parameter ratios *k* = *C*_2_/*C*_1_ and *r* = *L*_2_/*L*_1_. By maintaining *k* and *r* at constant values, the poles and zeros can be dynamically tracked through the adjustment of the capacitor values *C*_1_ and *C*_2_, as illustrated in Figure 4. In the implemented circuit, deviations from a constant capacitance ratio and the effects of parasitic elements can modify the frequency ratios.

**Figure 5:**
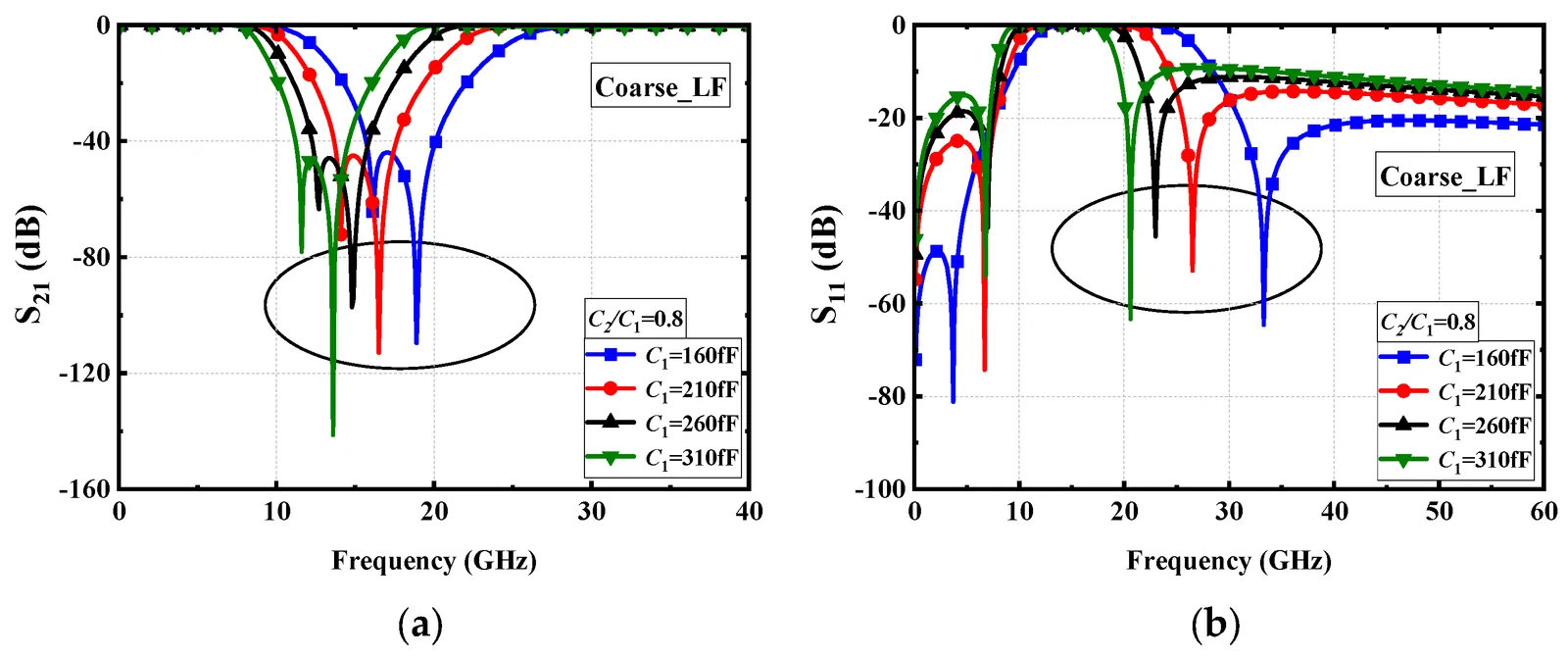
Simulated S_21_ and S_11_ characteristics of the proposed multi-mode reconfigurable filter under various *C*_1_ values in Coarse_LF mode with *k* = 0.8.

The transmission zeros (|S_21_| = 0) and transmission poles (|S_11_| = 0) of the proposed structure are determined by the following equations:

(15) $$S_{21}=\frac{2Z_{21}Z_{0}}{\left(Z_{11}+Z_{0}\right)\left(Z_{22}+Z_{0}\right)-Z_{12}Z_{21}}$$

(16) $$S_{11}=\frac{\left(Z_{11}-Z_{0}\right)\left(Z_{22}+Z_{0}\right)-Z_{12}Z_{21}}{\left(Z_{11}+Z_{0}\right)\left(Z_{22}+Z_{0}\right)-Z_{12}Z_{21}}$$

where Z_0_ denotes the port impedance, it can be derived from Equation (15) that the transmission zero frequencies correspond to the zeros of |Z_21_|. Although the transmission pole frequencies differ from the pole frequencies of |Z_21_|, they exhibit a strong correlation. Taking the Coarse_LF mode as an example, Figure 5 illustrates the simulated S-parameters of the proposed filter under varying values of *C*_1_ and *C*_2_. It is evident that as *C*_1_ varies, the transmission poles exhibit a variation trend similar to that of the poles in |Z_21_|.

### 2.3. Implementation of Variable Capacitor C

The preceding section analyzed in detail the pole–zero distribution and its tuning mechanism specifically, the continuous tuning of the pole and zero frequencies by adjusting variable capacitance; this section focuses on the actual implementation of the tunable capacitors and delves into their operating principles under both coarse- and fine-tuning modes.

**Figure 6:**
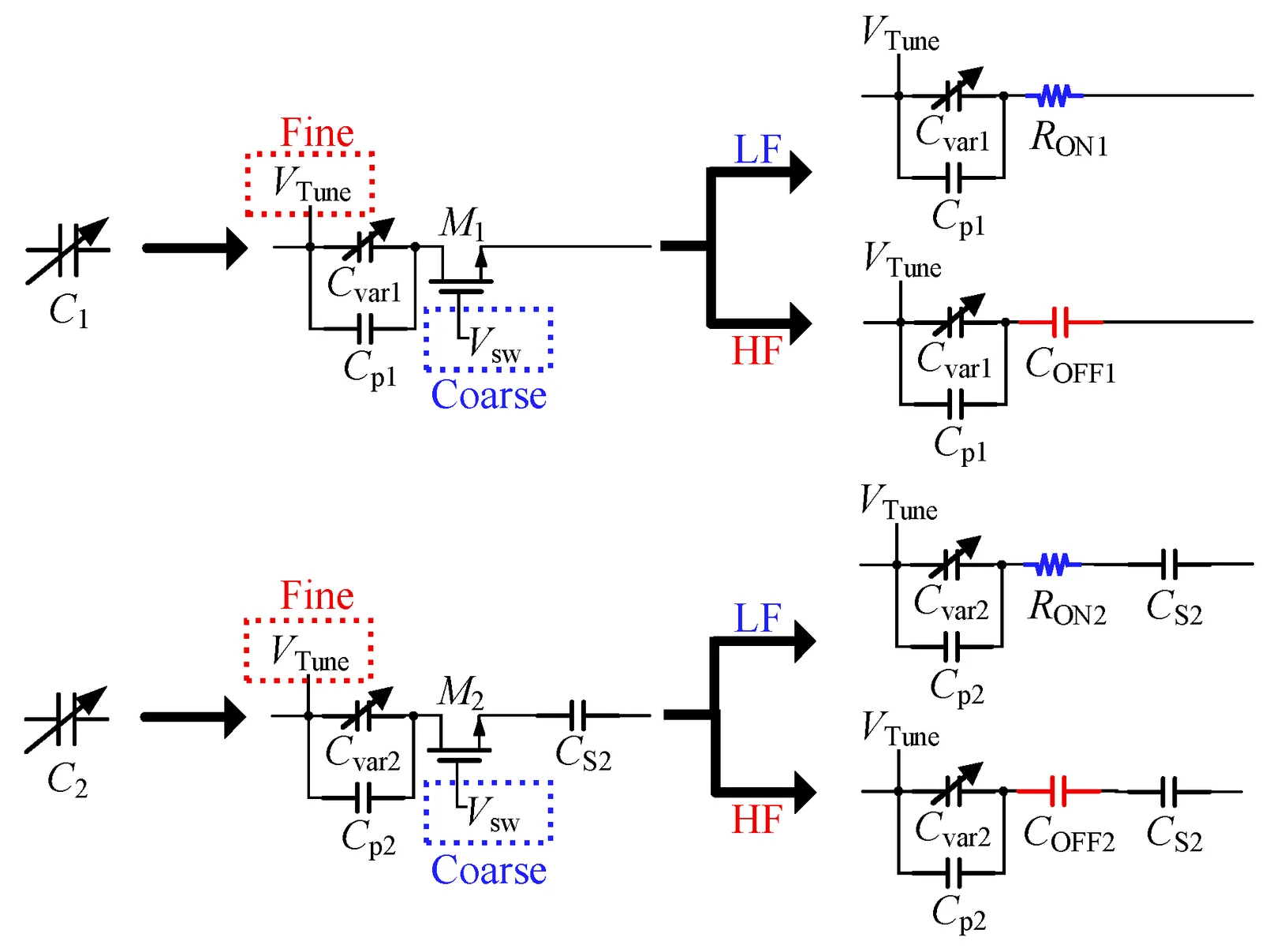
Schematic of the tunable capacitors.

As shown in Figure 6, the tunable capacitor *C*_1_ consists of a varactor *C*_var1_ in parallel with a capacitor *C*_P1_, which is further connected in series with a switching transistor *M*_1_. By adjusting the voltage *V*_SW_ to control the on/off state of *M*_1_, a wide-range transition between the low-frequency (LF) and high-frequency (HF) states can be achieved in the coarse mode. Meanwhile, by regulating the voltage *V*_Tune_ to vary the capacitance of the varactor *C*_var1_, the pole–zero tracking capability in the fine mode is realized. Compared to *C*_1_, the structural difference in *C*_2_ lies in an additional capacitor *C*_S2_ connected in series at the source of the switching transistor *M*_2_. This capacitor serves as a DC-blocking element to prevent the DC path from interfering with the switching states, thereby ensuring that the switching transistor can be effectively controlled by the external voltage. Notably, The fixed capacitors *C*_P1_, *C*_P2_, and *C*_S2_ are implemented using metal-oxide-metal (MOM) capacitors.

**Figure 7:**
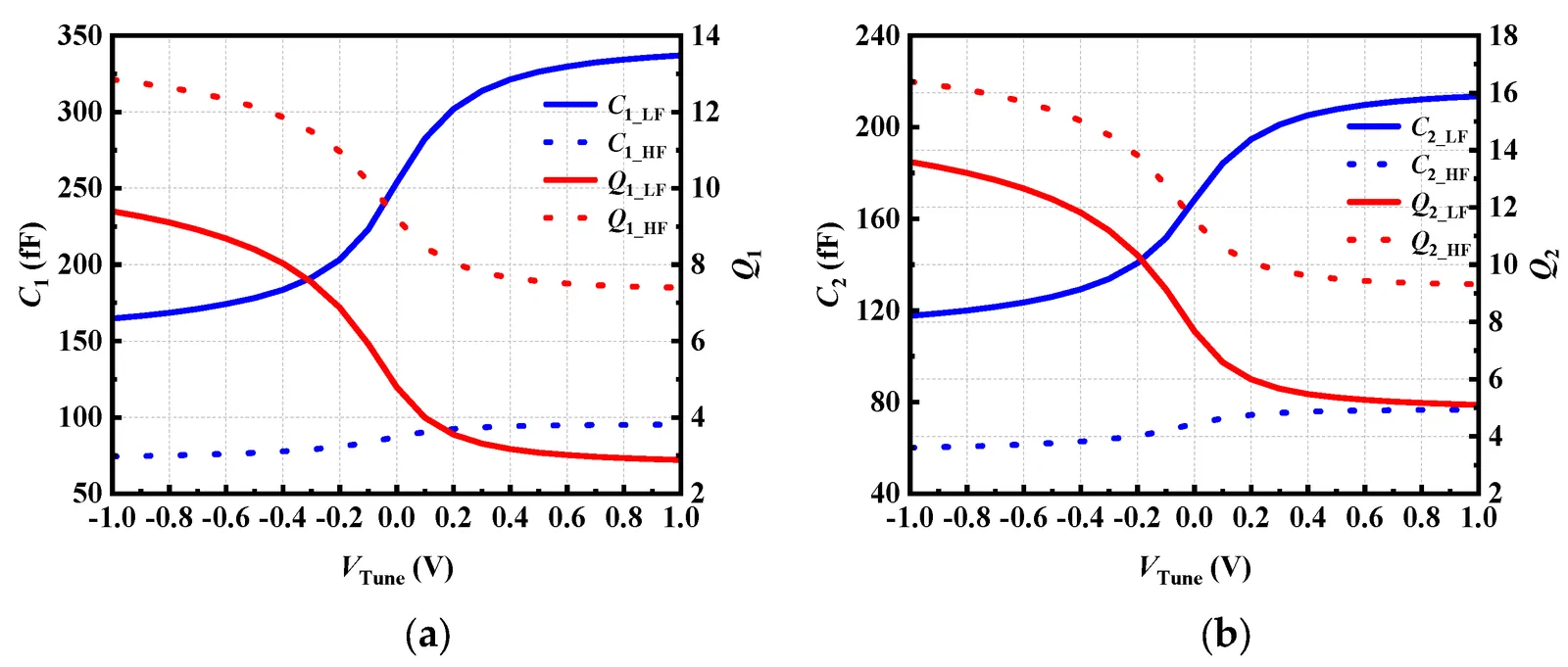
Capacitance values and *Q*-factors of the proposed tunable capacitors *C*_1_ and *C*_2_ in coarse and fine modes: (**a**) *C*_1_, (**b**) *C*_2_.

By comprehensively trading off the parasitic capacitances, on-resistances, capacitance values, and *Q*-factors of the transistors against the tuning range of the varactors, the optimized parameters of the tunable capacitors are determined, as presented in Figure 7.

Figure 7a and Figure 7b present the curves of the capacitance and quality factor (*Q*-factor) versus control voltages for the proposed tunable capacitors *C*_1_ and *C*_2_, respectively, under both coarse- and fine-tuning modes. The coarse-tuning mode, defined as the wide-range switching of the capacitance between the low-frequency (LF) and high-frequency (HF) states, is realized by toggling the switch control voltage *V*_SW_ between 0 and 1. Specifically, at *V*_Tune_ = −1 V, when *V*_SW_ switches from 0 to 1, the value of *C*_1_ abruptly drops from 165 fF to 74 fF, while *C*_2_ decreases from 118 fF to 60 fF. Under a fixed *V*_SW_ state, the fine-tuning mode enables continuous capacitance variation by dynamically sweeping the tuning voltage *V*_Tune_ from −1 V to 1 V with a step size of 0.1 V. As *V*_Tune_ increases from −1 V to 1 V, in the LF state (*V*_SW_ = 1), *C*_1_LF_ and *C*_2_LF_ continuously increase from 165 fF and 118 fF to 337 fF and 214 fF, respectively; in the HF state (*V*_SW_ = 0), *C*_1_HF_ and *C*_2_HF_ vary from 74 fF and 60 fF to 95 fF and 77 fF, respectively.

The lower *Q*-factor observed in the LF mode compared to the HF mode is primarily attributed to the on-resistances (*R*_ON1_ and *R*_ON2_) of the switch transistors. The gate width of the switch MOS transistors is optimized to 6 × 32 μm to mitigate the degradation of image rejection performance caused by large on-resistances.

The capacitance and Q-factor characteristics discussed above describe the steady-state behavior of the proposed tunable capacitors. The tuning speed of the proposed filter is mainly determined by the response speed of the external control-voltage source and its associated bias network. Within each coarse-tuning state, the pole and zero frequencies are tuned by varying *V*_Tune_, while *V*_SW_ remains fixed. Therefore, the proposed work focuses primarily on the pole–zero tuning mechanism and the resulting steady-state frequency responses, rather than on the design of the control-voltage driver. The actual tuning speed is mainly related to the charging and settling time of the external control source, together with the parasitic effects of the tuning network.

### 2.4. Impact of Quality Factor of the Inductors and Capacitors

**Figure 8:**
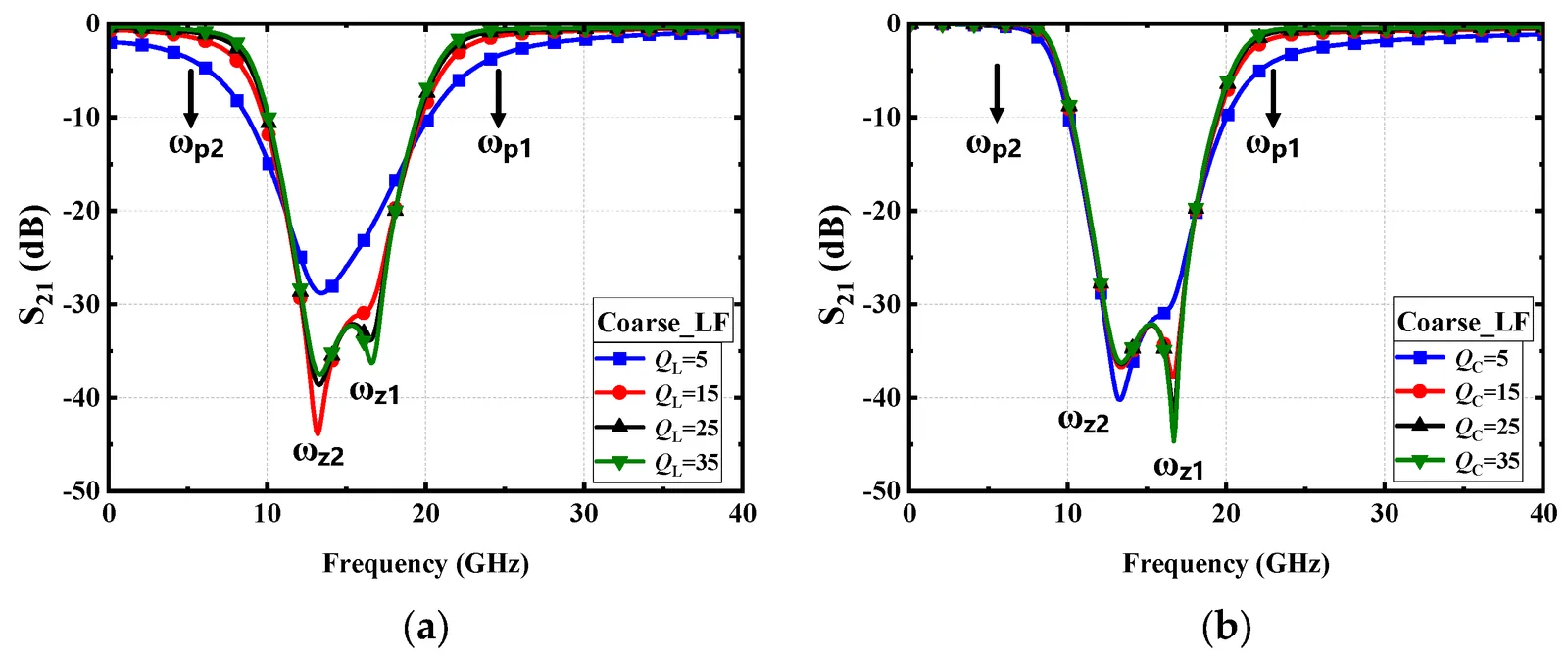
Simulated *S*_21_ responses of the proposed multi-mode reconfigurable filter in Coarse_LF mode as a function of (**a**) *Q*_L_ and (**b**) *Q*_C_.

The previous analysis was conducted under the assumption of lossless circuit elements to elucidate the distribution characteristics of the poles and zeros. To evaluate the impact of parasitic resistance while maintaining analytical simplicity, we assume that inductors *L*_1_ and *L*_2_ have the same quality factor *Q*_L_, and capacitors *C*_1_ and *C*_2_ also maintain the same quality factor *Q*_C_. Building upon these assumptions, this section further investigates the influence of *Q*_L_ and *Q*_C_ on the S21 responses in the Coarse_LF mode, as illustrated in Figure 8.

Figure 8a illustrates the simulated S21 responses as a function of the inductor quality factor *Q*_L_. It is observed that *Q*_L_ simultaneously impacts the passband insertion loss and the notch depths of the two transmission zeros. For optimal performance, the inductors are implemented using the thickest metal layer (*M*_9_) in the layout design. Figure 8b shows the simulated S21 curves with respect to the capacitor quality factor *Q*_C_. It is evident that *Q*_C_ primarily influences the loss at the high-frequency pole *ω*_p1_ and the notch depth of the high-frequency transmission zero *ω*_z1_. Furthermore, as indicated in Figure 7, when the filter operates in the Coarse_LF mode and the control voltage *V*_Tune_ is tuned from −1 V to 1 V with a step size of 0.1 V, the effective quality factors of the tunable capacitor networks *C*_1_ and *C*_2_ gradually degrade, resulting in a corresponding deterioration in the notch depth of the high-frequency transmission zero.

## 3. Multi-Mode Reconfigurable Filter Implementation and Measurement Results

**Figure 9:**
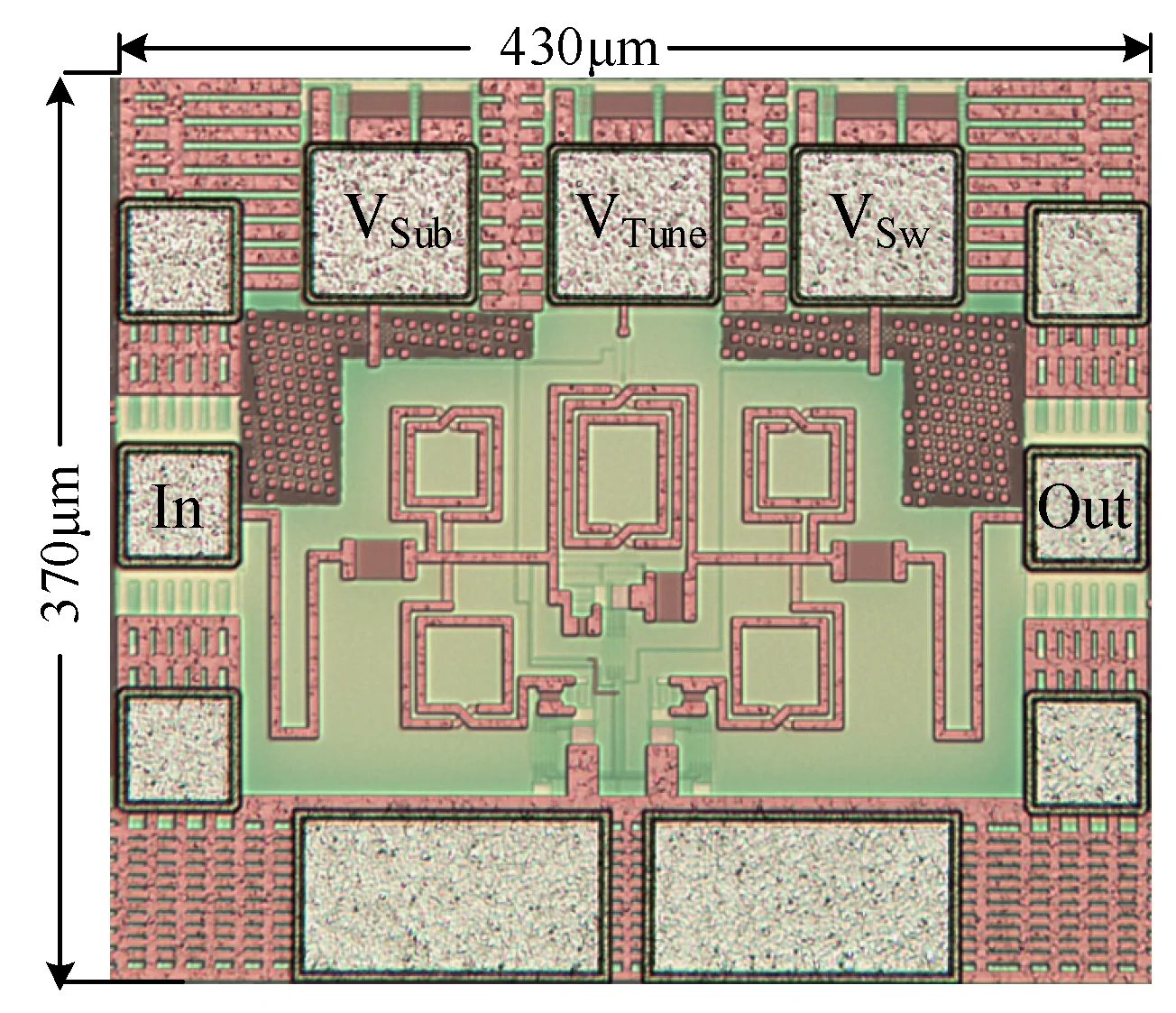
Multi-mode reconfigurable filter chip photo.

The proposed multi-mode reconfigurable filter was fabricated using the TSMC 65 nm CMOS process. Figure 9 shows the chip photo of the multi-mode reconfigurable filter, which occupies a core area of 320 μm × 200 μm. The total chip area, including the pads, is 430 μm × 370 μm.

**Figure 10:**
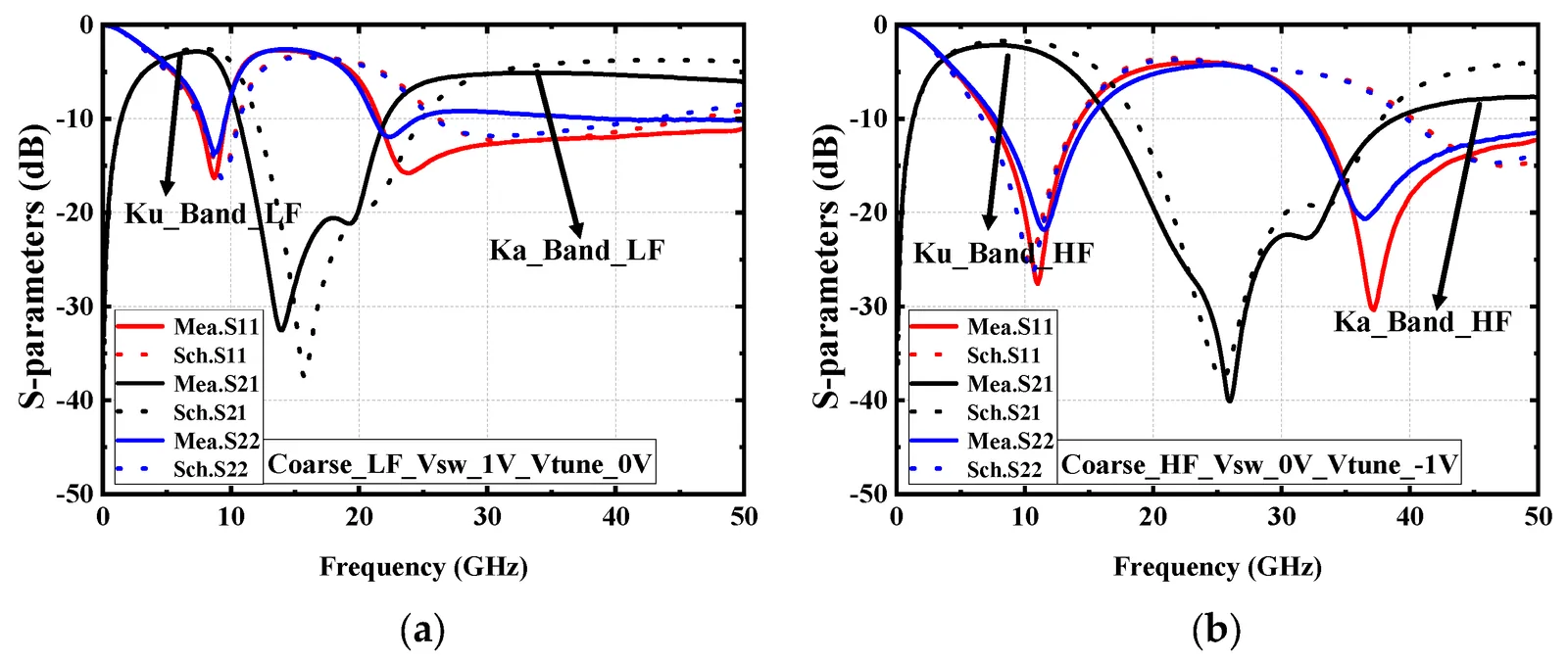

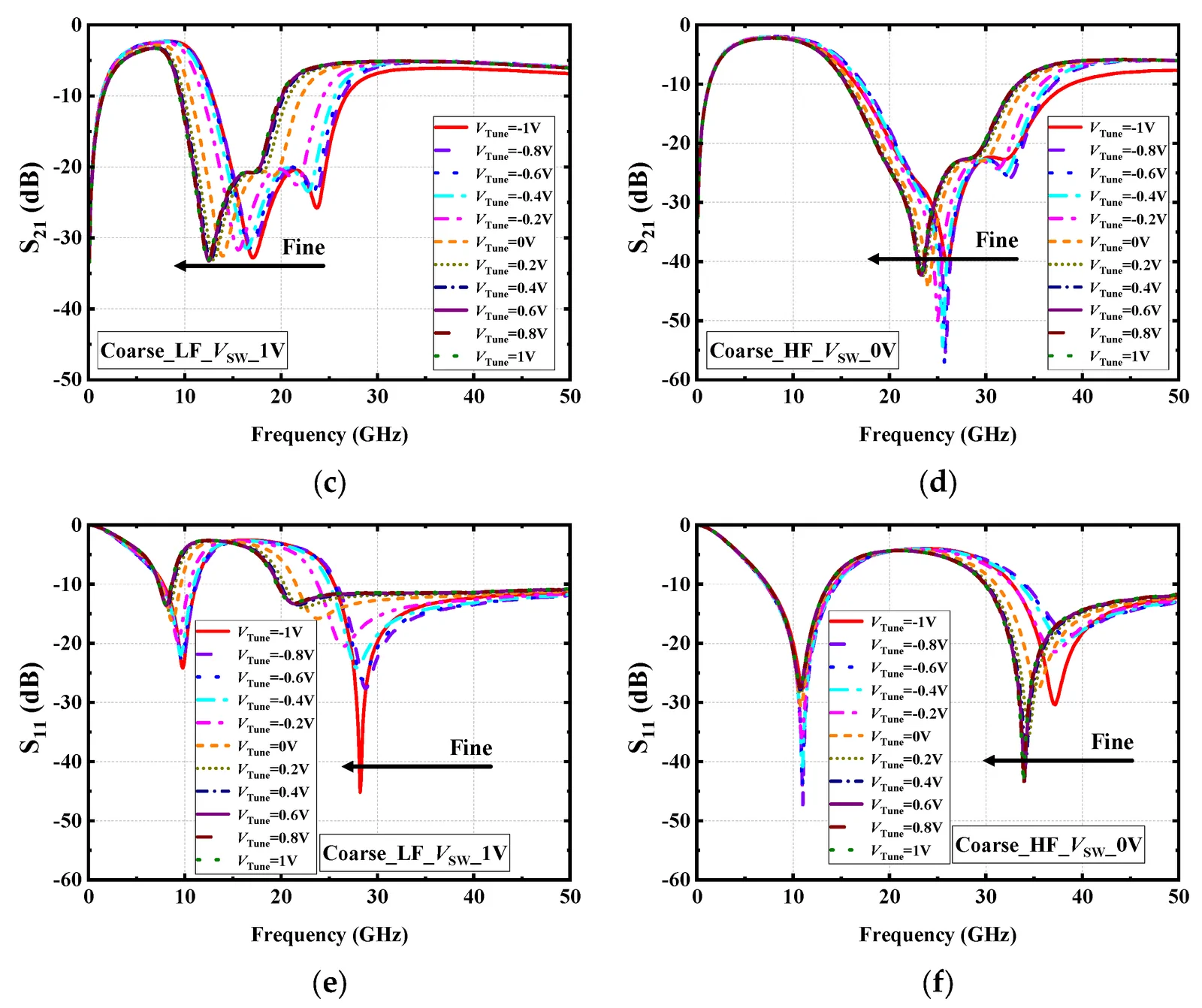
Simulated and measured S-parameters of the proposed filter under different tuning conditions. (**a**,**b**) Comparison of simulated and measured S11, S21, and S22 in the Coarse_LF mode with *V*_SW_ = 1 V and *V*_Tune_ = 0 V, and in the Coarse_HF mode with *V*_SW_ = 0 V and *V*_Tune_ = −1 V, respectively. (**c**,**d**) Measured S21 responses under fine-tuning in the Coarse_LF mode (*V*_SW_ = 1) and the Coarse_HF mode (*V*_SW_ = 0 V), respectively. (**e**,**f**) Measured S11 responses under fine-tuning in the Coarse_LF mode (*V*_SW_ = 1 V) and the Coarse_HF mode (*V*_SW_ = 0 V), respectively. In (**c**–**f**), *V*_Tune_ is swept from −1 to 1 V in steps of 0.2 V.

Figure 10a,b show the measured and simulated S-parameters of the proposed multi-mode reconfigurable filter under two representative states. In Coarse_LF mode (*V*_SW_ = 1 V, *V*_Tune_ = 0 V), the filter exhibits a stable bandstop response with a >15 dB rejection stopband covering 11.5–20.8 GHz (fractional bandwidth, FBW ≈ 57.6%), flanked by lower and upper passbands of 2.7–9.7 GHz and 24–29.5 GHz, respectively. In Coarse_HF mode (*V*_SW_ = 0 V, *V*_Tune_ = −1 V), the stopband is reconfigured to 18.6–35.2 GHz (FBW ≈ 61.7%) while maintaining >15 dB rejection, with the lower and upper passbands expanded to 2.7–13.8 GHz and 40–50 GHz. The excellent agreement between measured and simulated results validates the reliability of the proposed design in stopband reconfigurability and frequency selection. Within each selected coarse-tuning state, the proposed filter supports simultaneous signal transmission through the lower and upper passbands while rejecting signals in the intervening stopband. The Coarse_LF and Coarse_HF states are selected sequentially by *V*_SW_, whereas *V*_Tune_ provides continuous fine-tuning within each state. Thus, simultaneous dual-passband operation is supported within a fixed state, but the two coarse-tuning states cannot operate simultaneously.

Figure 10c–f illustrate the performance of the proposed reconfigurable filter under continuous tuning (Fine Mode), where the control voltage *V*_Tune_ is stepped from −1 V to 1 V with an increment of 0.2 V. In the Coarse_LF mode, the stopband rejection range (with >15 dB rejection) is continuously tuned from 14–25.3 GHz down to 10.2–18.7 GHz. Consequently, the lower passband is dynamically adjusted from 2.7 to 11.5 GHz, while the upper passband remains centered at 24–29.5 GHz. In the Coarse_HF mode, the tunable stopband is reconfigured from 18.6–35.2 GHz to 17.1–31.2 GHz, accompanied by the lower passband expanding up to 14.4 GHz and the upper passband tuning between 37 and 43.5 GHz. These results successfully demonstrate the high spectral flexibility and stopband reconfigurability of the proposed design. Simulation and experimental results demonstrate that the proposed reconfigurable filter has zero DC power consumption.

**Figure 11:**
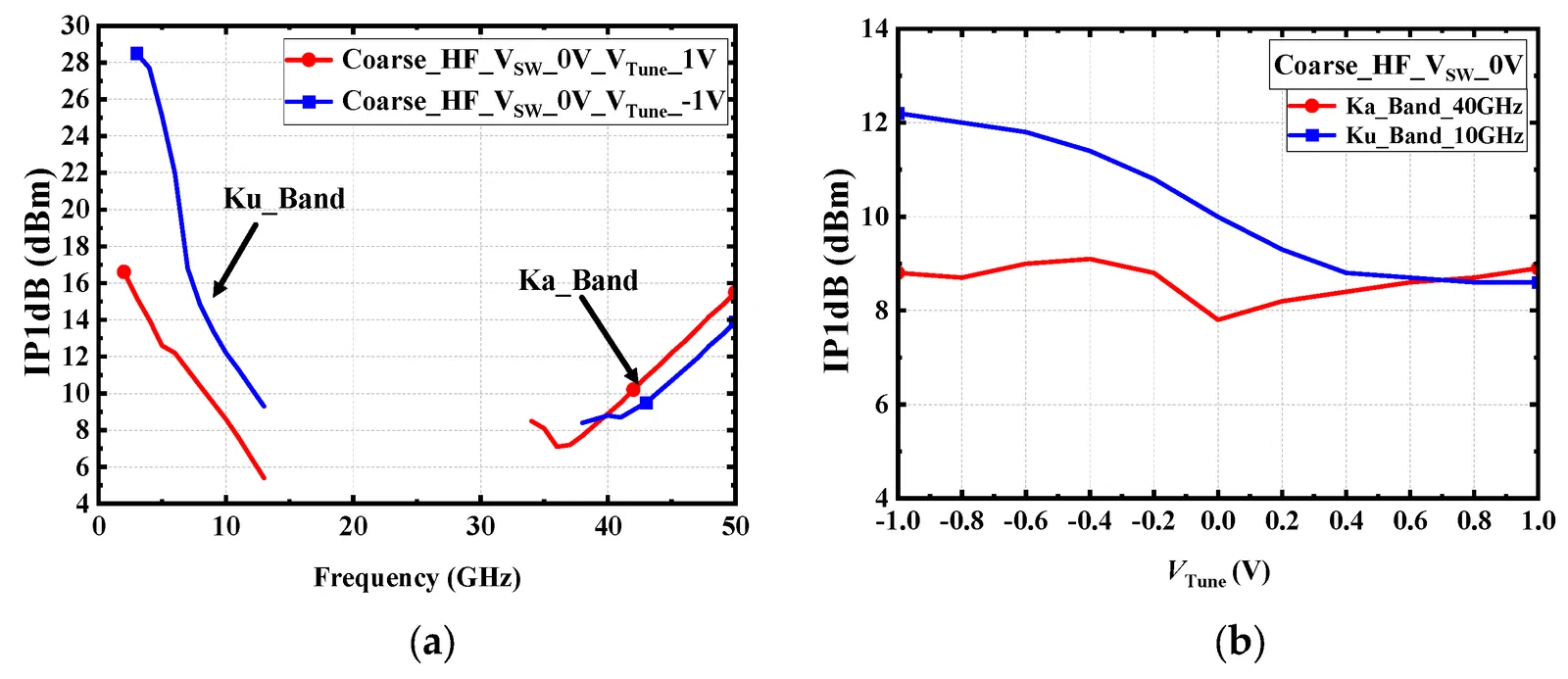
Measured input 1-dB compression point (IP1dB) of the proposed filter in Coarse_HF mode (*V*_SW_ = 0 V): (**a**) IP1dB versus frequency in the Ku and Ka-bands at *V*_Tune_ = −1 V and 1 V; (**b**) IP1dB versus fine-tuning voltage at 10 GHz and 40 GHz, with *V*_Tune_ swept from −1 V to 1 V in steps of 0.2 V.

As shown in Figure 11, the measured input 1-dB compression point (IP1dB) of the proposed filter varies with both frequency and tuning voltage in Coarse_HF mode (*V*_SW_ = 0 V). As shown in Figure 11a, in the Ku-band, the IP1dB decreases with increasing frequency. For *V*_Tune_ = 1 V, it decreases from 16.6 dBm at 2 GHz to 5.4 dBm at 13 GHz, whereas for *V*_Tune_ = −1 V, it decreases from 28.5 dBm at 3 GHz to 9.3 dBm at 13 GHz. In the Ka-band, the IP1dB generally increases with frequency after reaching a minimum value. It varies from 7.1 to 15.5 dBm over 34–50 GHz for *V*_Tune_ = 1 V, and from 8.4 to 13.9 dBm over 38–50 GHz for *V*_Tune_ = −1 V. Figure 11b further illustrates the influence of fine-tuning on the IP1dB. At 10 GHz, the IP1dB decreases from 12.2 to 8.6 dBm as *V*_Tune_ increases from −1 to 1 V. At 40 GHz, it varies within a relatively narrow range of 7.8–9.1 dBm, reaching its minimum near *V*_Tune_ = 0 V. These results demonstrate that the IP1dB is strongly dependent on the operating band and frequency, while its variation with fine-tuning voltage is more pronounced in the Ku-band than in the Ka-band. In addition, in Coarse_LF mode (*V*_SW_ = 1 V), the IP1dB exceeds 20 dBm over both 3–12 GHz and 27–50 GHz at *V*_Tune_ = 1 V.

To evaluate its performance, Table 1 summarizes the key characteristics of the proposed multi-mode reconfigurable filter in comparison with other state-of-the-art designs reported in the recent literature. Compared to references [13,14,15,16,17], which generally utilize fixed or single-stage tuning mechanisms, the proposed circuit uniquely incorporates a dual coarse- and fine-tuning mechanism. This approach enables enhanced dynamic reconfigurability of the stopband frequencies, allowing precise and flexible control over filtering characteristics. Furthermore, when compared to the design presented in [19], the proposed filter demonstrates superior performance by achieving a wider 15-dB stopband fractional bandwidth (15-dB SB FBW) and greater maximum attenuation (Max. Att.), which are critical for effective interference suppression. Benefiting from the distinctive transmission pole and zero distribution inherent to the filter topology, the structure attains exceptional stopband rejection across the targeted Ku- and Ka-band frequencies, making it highly suitable for advanced RF and millimeter-wave applications.

## 4. Conclusions

A compact, multi-mode reconfigurable filter implemented in a 65-nm CMOS process is presented for Ku/Ka multi-band applications. A pole–zero tuning scheme combining coarse and fine control is utilized. By employing lumped-element LC series and parallel resonant networks, two poles and two transmission zeros are generated, with the zeros inherently clamped between the poles due to the filter topology. The proposed design features a compact footprint, a wide stopband, and pole–zero tracking capabilities. Good agreement is obtained between the simulation and measurement results. The proposed filter structure can be a candidate for receiver systems that require wideband interference rejection, compact footprint, and continuous tunability.

## Figures and Tables

**Table 1:** Performance comparison with other state-of-the-art filters.

Ref.	Technology	Mode	fcs/T.R. (GHz)	15-dB SB FBW	Max. Att. (dB)	fcp (GHz)	3-dB BW (GHz)	IL (dB)	RL-Max dB)	Size (mm^2^)
[13]	0.13-μm BiCMOS	-	59.4 /Static	6.7%+	20	35.3	7.7	1.7	25+	0.039
[14]	0.13-μm BiCMOS	-	67 /Static	18.2%+	25	40	8.2+	1.7	19	0.012
[15]	0.13-μm BiCMOS	-	24.5 /Static	6% *	23.1	45	-	0.95	24	0.038
[16]	0.18-μm CMOS	-	53.2 /Static	5.6% *	14.2	39.8	-	0.5+	23.1	0.024
[17]	0.18-μm CMOS	-	30, 85 /Static	37%, 11%	25,18	55	-	4.6+	20	0.6
			30, 80 /Static	39%+, 10%+	35,25	55	-	2.5	50	
[19]	65-nm CMOS	-	24.9–28.7 /14.2%	40.2%	32.1–35	36.5–38+	4–7	2.7	17	0.036
			24.6–29.8 /19.1%	38.2%	21.6–29.5	36.5–38.8+	2.5–7	3.5	33	
[20]	GaAs IPD	-	29.5 /Static	16%	42	21/36	-	0.5	20	0.9
[21]	VO_2_	Work1	-	-	-	30.1–36.5	22–22.3	2.7–4.4	20	0.184
		Work2	-	-	-	38.5–49	4.7–6	1.4–3	30	0.51
This work	65-nm CMOS	LF	14.5–19.7 /30.4%	58%	31.4–33.3	5.7–7.2@Ku 35.4–39@Ka	5.9–8.7@Ku 22.7–29.2@Ka	2.3–6.3@Ku 5.1–9.1@Ka	24.2@Ku 27.6@Ka	0.064
		HF	24.1–26.9 /11%	61.7%	40.1–58.2	7.9–8.6@Ku 42–44@Ka	10.4–11.7@Ku 12–16@Ka	2–5.2@Ku 5.8–10.6@Ka	44@Ku 42.3@Ka	

T.R.: tuning range; SB: stopband; Att: attenuation; fcs: stopband center frequency. fcp: passband center frequency; RL-Max: maximum passband return loss value; 3-dB BW: 3-dB passband bandwidth. *: 10-dB stopband FBW; +: evaluated graphically.

## Data Availability

Data are contained within the article.
